# Pharmacokinetic and Pharmacodynamic Characteristics of Pelubiprofen Tromethamine vs. Pelubiprofen in Healthy Subjects

**DOI:** 10.3390/pharmaceutics15041280

**Published:** 2023-04-19

**Authors:** Yu-Jeong Son, Min-Kyu Park, Hyeon-Jeong Park, Ha-Yeon Kim, Ye-Lim Jang, Young-Sim Choi, Jun-Gi Hwang, Ji-Hyung Seo, Yu-Kyong Kim

**Affiliations:** 1 Pharmacology Major in Department of Medicine Graduate School, Chungbuk National University, Cheongju 28644, Republic of Korea; son1079@naver.com (Y.-J.S.); mk_park@cbnuhctc.com (M.-K.P.); 2 Department of Clinical Pharmacology and Therapeutics, Chungbuk National University College of Medicine and Hospital, Cheongju 28644, Republic of Korea; hj_park@cbnuhctc.com (H.-J.P.); hy_kim@cbnuhctc.com (H.-Y.K.); yl_jang@cbnuhctc.com (Y.-L.J.); ys_choi@cbnuhctc.com (Y.-S.C.); jk_hwang@cbnuhctc.com (J.-G.H.); 3 Daewon Pharmaceutical Co., Ltd., Seoul 04808, Republic of Korea; jhseo@daewonpharm.com

**Keywords:** phase I, comparative pharmacokinetics/pharmacodynamics, tromethamine salt, COX-2 inhibitory effect

## Abstract

Compared to pelubiprofen, a cyclooxygenase-2-selective inhibitor, pelubiprofen tromethamine has been reported to exhibit improved solubility and absorption. Pelubiprofen tromethamine combines the anti-inflammatory effect of pelubiprofen with the gastric protective function of tromethamine salt, making it a relatively safe class of non-steroidal anti-inflammatory drugs with low levels of gastrointestinal side effects in addition to its original analgesic, anti-inflammatory, and antipyretic effects. This study assessed the pharmacokinetic and pharmacodynamic characteristics of pelubiprofen and pelubiprofen tromethamine in healthy subjects. Two independent clinical trials were performed in healthy subjects using a randomized, open-label, oral, single-dose, two-sequence, four-period, crossover design. In Study I and Study II, subjects received 25 mg of pelubiprofen tromethamine and 30 mg of pelubiprofen tromethamine, respectively, with 30 mg of pelubiprofen being the reference. Study I fell within the bioequivalence study criteria. A trend of increased absorption and exposure for 30 mg of pelubiprofen tromethamine vs. the reference in Study II was observed. The maximum cyclooxygenase-2 inhibitory effect of 25 mg of pelubiprofen tromethamine was approximately 98% compared to the reference, showing no significant pharmacodynamic variation. It is thus predicted that 25 mg of pelubiprofen tromethamine would show no clinically significant discrepancies in clinical analgesic and antipyretic effects from 30 mg of pelubiprofen.

## 1. Introduction

Non-steroidal anti-inflammatory drugs (NSAIDs) are some of the most commonly prescribed drugs worldwide, with a wide spectrum of clinical utility in the management of pain, fever, and inflammatory conditions [1,2]. NSAIDs exhibit pharmacological effects by inhibiting cyclooxygenase (COX) activity and reducing the production of prostaglandin E_2_ (PGE_2_), which are known to initiate pain, fever, and inflammation. The inhibition of COX, through the action of NSAIDs, reduces the formation of not only PGE_2_, but also prostaglandin D_2_ (PGD_2_), prostaglandin F_2_ (PGF_2_), prostaglandin I_2_ (PGI_2_), and thromboxane A_2_ (TXA_2_) [3]. COX is broadly divided into COX-1, which affects physiological functions such as the protection of the gastric mucosa, and COX-2, an inducible type in inflammatory responses [4]. Most NSAIDs non-selectively inhibit COX-1 and COX-2. As a result, approximately 40% of users taking NSAIDs experience upper gastrointestinal symptoms as a major adverse event of NSAIDs caused by COX-1 inhibition. Approximately 1–2% of users experience a severe gastrointestinal complication [5]. To resolve this issue, COX-2-selective inhibitors were developed, and studies on the COX-2-related anticancer activity are actively ongoing [6,7,8]. The key composition of COX-2-selective inhibitors includes celecoxib and rofecoxib. Related to their pharmacological mechanisms, COX-2-selective inhibitors exhibit anti-inflammatory, antipyretic, and analgesic effects, while showing relatively low levels of potential gastrointestinal side effects [6,7,8,9].

Pelubiprofen is a well-known COX-2-selective inhibitor and a 2-arylpropionic acid drug with little effect on gastro-intestinal disorders [10,11]. Upon oral administration, pelubiprofen can selectively inhibit COX-2, via the mechanism that reduces the PGE_2_ concentration, which is expected to be related to the elicitation of anti-inflammatory, antipyretic, and analgesic activities. In addition, as salt formation is the most common method for increasing drug solubility, one salt compound, tromethamine, acts as a proton acceptor to bind to H^+^ in the body for the unique function of acidosis correction and prevention [12]. Tromethamine is well-known as “Tris buffer” (in gel electrophoresis buffers, etc.) [13]. Accordingly, the fusion of tromethamine salt with pelubiprofen was expected to enhance drug safety, solubility, and absorption in accordance with a previous study performed by one of the researchers (Park, M.K.) of the present study [14]. Therefore, pelubiprofen tromethamine combines the anti-inflammatory effect of pelubiprofen with the gastric-protective function of tromethamine salt, making it a relatively safe class of NSAIDs with low levels of gastrointestinal side effects on top of its original analgesic, anti-inflammatory, and antipyretic effects.

The fusion of tromethamine salt increases the bioavailability of the drug pelubiprofen, which increases the exposure to the body compared to the already approved 30 mg dose of pelubiprofen [14]. For this reason, when a new formulation of pelubiprofen tromethamine is developed, the dose should be validated for the equivalent level of exposure to the conventional form. Therefore, this study assessed the pharmacokinetic and pharmacodynamic characteristics of pelubiprofen and pelubiprofen tromethamine in healthy subjects.

## 2. Materials and Methods

### 2.1. The Investigational Drug, Ethics Approval, and Consent to Participate

This study was conducted with healthy adult subjects who are 19 years or older, weighing ≥ 50.0 kg, and with a BMI ranging between ≥18.0 kg/m^2^ and ≤30.0 kg/m^2^. The suitability of subjects was decided in accordance with the clinical laboratory test results regarding hematology, blood chemistry, urine, urine drug screening, serology test, etc., which were set and performed according to the characteristics of the investigated drug. In addition, the results of the vital signs, physical examination, and 12-lead electrocardiogram (ECG) assessments were considered.

The exclusion criteria were as follows: a history of clinically significant hypersensitivity events to the investigational drug component, adjunct, and other drugs; testing positive for drug abuse following a urine drug screening or a history of drug abuse; gastrointestinal disease or related surgery that may affect drug absorption; having smoked ten or more cigarettes a day within the three months preceding the first administration. All subjects provided informed consent before participation in any procedure.

The study protocol, including the informed consent form, was approved by the Chungbuk National University Hospital Institutional Review Board (IRB), and conducted at the Clinical Trials Center, Chungbuk National University Hospital, Cheongju, Republic of Korea (ClinicalTrials.gov registry no.: NCT05214690, NCT05214677). The IRB approval numbers for Study I on 25 mg of pelubiprofen tromethamine vs. 30 mg of pelubiprofen and for Study II on 30 mg of pelubiprofen tromethamine vs. 30 mg of pelubiprofen were 2020-06-007 and 2020-06-006, respectively. The IRB approval number for the associated pharmacodynamic evaluation in Study I was 2020-06-023.

In addition, to prioritize the subjects’ rights and safety in all three studies, the study was conducted in compliance with the ethical regulations of the International Council for Harmonisation of Technical Requirements for Pharmaceuticals for Human Use (ICH) guideline and the Helsinki Declaration (Fortaleza, Brazil, 2013) as well as the Korean Good Clinical Practice (KGCP), along with the domestic Standard Operating Procedure (SOP).

### 2.2. Study Design

**Figure 1:**
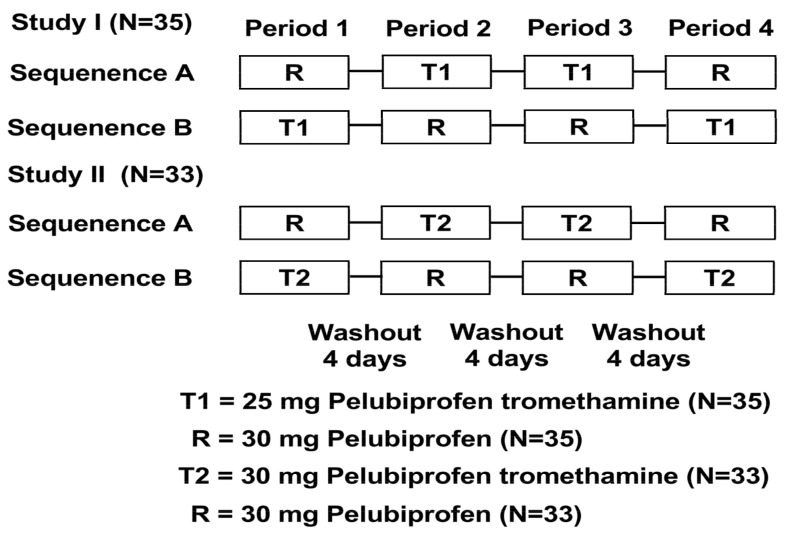
Study design.

In this study, the two independent clinical trials (Study I and II) were conducted using an identical randomized, open-label, oral, single-dose, fasted, two-sequence, four-period, crossover design. Due to the known pharmacokinetic characteristics of pelubiprofen with its large intra-subject variability, the replicated design was chosen to calculate the true intra-coefficient of variation in both studies. Study I tested 25 mg of pelubiprofen tromethamine (36.73 mg of pelubiprofen tromethamine salt; 25 mg of pelubiprofen, Daewon Pharmaceutical Co., Ltd., Seoul, Republic of Korea) against 30 mg of pelubiprofen (30.0 mg of pelubiprofen, Daewon Pharmaceutical Co., Ltd., Seoul, Republic of Korea) as the reference. Study II tested 30 mg of pelubiprofen tromethamine (44.07 mg of pelubiprofen tromethamine salt; 30 mg of pelubiprofen, Daewon Pharmaceutical Co., Ltd., Seoul, Republic of Korea) against 30 mg of pelubiprofen (30.0 mg of pelubiprofen, Daewon Pharmaceutical Co., Ltd., Seoul, Republic of Korea) as the reference. The target number of subjects in both Study I and II was set to N = 36 (N = 18 for each sequence group) (Figure 1, Table 1).

The subject screening in both Study I and Study II was performed in the four weeks (Day 28 to Day 1) prior to the first administration (Day 1). When a screened subject had to drop out of the study after randomization and prior to first administration of the investigational drug owing to other reasons, the substitute subject was assigned a new number. In addition, the screened subjects fasted for at least 10 h, excluding drinking water, from approximately 10 p.m. until administration of the investigational drug. The randomization in both Study I and II led the subjects to receive oral administration of a single test or reference tablet with approximately 150 mL of water at each time point from approximately 8 a.m. at approximately 2 min intervals according to their numbers. The subjects in both Study I and II were prohibited from drinking water for an hour before and after the administration, except during drug administration. The post-administration blood sampling and tests were performed as scheduled. All subjects were provided with identical meals approximately 4 h after blood sampling. The subjects in both Study I and II received the blood sampling and tests for the pharmacokinetic (PK) evaluation approximately 8 h after administration. Then, they were discharged following the completion of the first period of the study.

To analyze the PK characteristics in both Study I and II, approximately 5 mL of blood was sampled before administration (0 h) and at the following time points after administration; 0.083 h (5 min), 0.167 h (10 min), 0.33 h (20 min), and 0.5, 0.75, 1, 1.5, 2, 2.5, 3, 3.5, 4, 6, and 8 h. Therefore, for each subject, the blood sampling was performed 15 times in each period, resulting in a total of 60 times. All four periods proceeded on the same schedule as the first period in both Study I and II. The wash-out period between each consecutive period was set to a minimum of four days. The subjects in both Study I and II, after completing all four periods, chose a day from Day 5 to Day 7 of the final administration in the four period for visiting the center to undergo post study visit.

In both Study I and II, the clinical study protocol was followed to complete the blood sampling for pharmacokinetic (PK) analysis. In both Study I and II, the safety evaluation was performed on subjects administered with the investigational drug at least once. Any clinically significant findings in the interview, physical examination, vital signs, 12-lead electrocardiogram (ECG), the clinical laboratory test, etc., during the study period were collected as adverse events, which were subsequently evaluated for the incidence of serious adverse events, severity, causal relationship, etc. All relevant data were collected through monitoring by the investigator and voluntary reporting by the subjects. To collect additional data, interview and physical examination were performed where necessary. The investigator carried out the follow-up monitoring of the subjects with adverse events until the symptoms subsided and abnormal test values returned to a healthy baseline or until a satisfactory explanation for the observed change could be provided.

To analyze the pharmacodynamic (PD) characteristics, approximately 1 mL of blood was sampled from the subjects in Study I, who had agreed to participate in the associated PD evaluation. The blood sampling was performed before administration (0 h) and at the following time points after administration: 0.083 h (5 min), 0.167 h (10 min), 0.33 h (20 min) and 0.5, 0.75, 1, 1.5, 2, 2.5, 3, 3.5, 4, 6, and 8 h. Therefore, for each subject, the blood sampling was performed 15 times in each period. While the PD analysis was performed on the same schedule as the PK analysis, only period 1 and 2 results were included in the analyses, and the post study visit was not performed.

### 2.3. Dose Determination

The dosage regimen of the test in Studies I and II was established in accordance with the dosage regimen of the reference pelubiprofen tablet (30.0 mg of pelubiprofen, Daewon Pharmaceutical Co., Ltd., Seoul, Republic of Korea). For Study II, the dosage of the test product was set to 30 mg, which is the approved dose. For Study I, taking the change in exposure of pelubiprofen due to tromethamine into consideration, 25 mg was tested. A single tablet of test and reference products were administered 3 times a day after meal.

### 2.4. Bioanalytical Method Validation of Study I and II

The validation of the quantitative analysis of plasma pelubiprofen in this study was performed in accordance with the following guidelines: the Validation Protocol for Pelubiprofen Quantification, the domestic Standard Operating Procedure (SOP) for Bioanalyses, the Guideline of Bioanalytical Method Validation (MFDS, December 2013), and Bioanalytical Method Validation Guidance for Industry (USA FDA, 2018). The plasma pelubiprofen was pretreated using protein precipitation in a dark room at room temperature (15–25 °C) and under shielded light for the subsequent liquid chromatograph tandem mass spectrometry (LC-MS/MS) analysis. The tested items in the validation included the intra-batch and inter-batch accuracy, precision, selectivity, linearity, and stability.

The results of intra-batch and inter-batch tests at the lower limit of quantification (LLOQ) had an accuracy ranging from 85 to 115% (LLOQ; 80–120%) and precision was <15% (LLOQ; <20%) to satisfy the pre-determined criteria. The validation result of the Signal-to-Noise ratio (S/N ratio) at LLOQ was ≥5. The analytic target substance was separated from interfering substances in biological samples. The calibration curve was drawn at the 0.5–1000 ng/mL range of plasma concentration, and an acceptable linearity with the correlation coefficient (r) ≥ 0.9950 was observed. The stability of the analytic target substance satisfied the criteria of 85–115% accuracy for each concentration. The stability of the whole blood was within the ±1% range of the change % for the test and reference samples.

### 2.5. Pharmacokinetic Analysis

In both Study I and II, the clinical study protocol was followed to complete the blood sampling for pharmacokinetic (PK) analysis according to the schedule after the administration of investigational drug. In both Study I and II, blood was sampled in a sodium heparin tube (or a syringe). After 10 min centrifugation (4 °C, 1836× *g*) of the sampled blood, the plasma was transferred to two 2 mL polypropylene tubes and stored in a freezer (approximately −70 °C) for subsequent analyses.

In both studies, liquid chromatograph tandem mass spectrometry (LC-MS/MS) analysis conditions were established for the analysis of plasma pelubiprofen by referring to the previously reported analysis method. In addition, after completing validation for accuracy, precision, selectivity, linearity, etc., using LC-MS/MS as per the in-house full validation report produced, it was applied to sample analysis. There were no re-analyses in either Study I or II. The PK analysis was subsequently performed on the subjects exhibiting a quantifiable plasma drug concentration. The effect time in accordance with concentration regarding the PK parameters was based on the time of blood sampling (actual time) after the investigational drug administration. The plotting mean plasma concentration against time for the investigational drug was represented as a linear or log-linear graph. In addition, the PK parameters were estimated at the concentrations measured after the investigational drug administration.

If the concentration at a certain time point displayed a value below the lower limit of quantification (LLOQ), the value before the time of peak plasma concentration (T_max_) was set to “0” and the subsequent value was set to “missing” or “blank” for the PK analysis performed using the Phoenix WinNonlin^®^ Ver. 8.2 (Pharsight, CA, USA) and following the SOP of the respective institution and the general guideline of PK analysis. Next, the numerical values of PK parameters were obtained for each subject, and the mean and standard deviation were presented for each drug administration sequence group in descriptive statistics. The Phoenix WinNonlin^®^ Ver. 8.2 (Pharsight, CA, USA) was used to convert the log transformed values of the PK parameters of plasma pelubiprofen in each drug administration sequence group; maximum concentration (C_max_), the area under the time vs. concentration curve from time 0 to the last measurable timepoint (AUC_0-t_), and the area under the time vs. concentration curve from time 0 to infinity (AUC_0-inf_) were included into the geometric mean ratios (GMR) and the 90% confidence interval (90% Cl).

### 2.6. Pharmacodynamic Analysis

The pharmacodynamic (PD) analysis was performed on the subjects in Study I, who had agreed to participate in the PD evaluation. For pharmacodynamic evaluation, the lipopolysaccharide (LPS) from E. coli O111:B4 (Sigma-Aldrich, Seoul, Republic of Korea) and the prostaglandin E_2_ (PGE_2_) enzyme-linked immunosorbent assay (ELISA) monoclonal kit (Cayman Chemical, MI, USA) were used. This study was conducted on healthy subjects, and LPS was added to sampled blood to induce inflammatory gene expression. The following procedures were applied for the PGE_2_ concentration analysis of subjects in Study I, who had agreed to participate in the pharmacodynamic evaluation.

Into 1.0 mL of sampled blood, 10 μL of lipopolysaccharide (LPS) was added. The mixture was centrifuged at 3000 1836× *g* and 4 °C for 10 min. By mixing the PGE_2_ ELISA standard and 1.0 mL of ELISA buffer, the standard reagents were produced at 1000, 500, 250, 125, 62.5, 31.3, 15.6, 7.8 pg/mL concentrations. Subsequently, to each well of the 96-well plate, 50 μL of the standard reagent, PGE_2_ acetylcholinesterase (AChE) tracer, and PGE_2_ monoclonal antibody were added. Each plate was then covered with a plastic film to be cultured at 4 °C for 18 h. Then, the wells were washed with the buffer solution 5 times, after which 200 μL of Ellman’s reagent was added to each well. The plate was covered with film once more to be placed on an orbital shaker for 60 min. The plate was read at a wavelength of 410 nm.

Pharmacodynamic (PD) was analyzed with data from the first and second periods. The plasma Prostaglandin E_2_ (PGE_2_) concentration in the first and second periods of Study I was measured using the enzyme-linked immunosorbent assay (ELISA) at the same time points as in the PK analysis: before investigational drug administration (0 h) and after drug administration at 0.083 h (5 min), 0.167 h (10 min), 0.33 h (20 min), and 0.5, 0.75, 1, 1.5, 2, 2.5, 3, 3.5, 4, 6, and 8 h. Therefore, for each subject, the measurements were taken 15 times in each period. While the PD analysis was performed on the same schedule as the PK analysis, only period 1 and 2 results were included in the analyses, and the post-study visit was not performed.

The PGE_2_ concentration and the cyclooxygenase-2 (COX-2) inhibitory effect derived from baseline (0 h time point before drug administration) values for each subject in Study I were visualized. The decreased level of calibrated PGE_2_ concentration performed in accordance with the concentration at baseline for each subject was tested as the extent of COX-2 inhibition, and the GMR and respective 90% CI of the COX-2 inhibition were applied in the PD analysis.

### 2.7. Statistical Analysis

#### 2.7.1. Pharmacokinetic Data

In both Study I and II, the pharmacokinetic (PK) statistical analysis was performed using the SAS^®^ Analytics Pro Ver. 9.4 (SAS Institute Inc., Cary, NC, USA). Without a specific reference, the significance level in all statistical analyses was set to 5% and a two-sided test was performed in principle. The numerical values of PK parameters were obtained for each subject, and the mean and standard deviation of each drug administration sequence group were presented in descriptive statistics. Based on 30 mg of pelubiprofen, the GMR for pelubiprofen tromethamine was calculated, and the 90% CI for it was calculated. In this study, the calculated GMR and 90% Cl were compared.

#### 2.7.2. Pharmacodynamic Data

The pharmacodynamic (PD) data from the subjects in Study I, who had agreed to participate in the PD evaluation, were analyzed as follows to check for outliers. Grubbs’ test was performed when the normality of the data was satisfied; otherwise, a boxplot was drawn for log-transformed values, after which values outside the interquartile range (IQR) were checked for subsequent use in Grubb’s test. For significant outliers, the data excluding the values were analyzed once again.

## 3. Results

### 3.1. Demographic Characteristics

#### 3.1.1. Study I: Study of 25 mg of Pelubiprofen Tromethamine vs. 30 mg of Pelubiprofen

An explanation of the study was given to 61 subjects, after which screening, including vital signs, physical examination, 12-lead electrocardiogram (ECG), the clinical laboratory test, etc., was performed. On the day of admission in the first period of the study, 36 subjects were each given a subject number. However, as one of them had to drop out, as determined by the principal investigator prior to the first administration of the investigational drug, a new subject was recruited for replacement, so subject numbers up to 37 were used for randomizing the healthy subjects. Later, one more subject dropped out in the fourth period of the study, as determined by the principal investigator performed in accordance with the test results on the day of admission prior to the drug administration. Hence, 36 subjects received at least one administration of the investigational drug, whereas 35 subjects completed the entire clinical trial (Figure 1).

The mean values of age, weight, height, and BMI were 25.41 years, 74.85 kg, 173.04 cm, and 24.96 kg/m^2^, respectively, for the 37 randomized subjects. The variation in age across the subjects assigned to each sequence group was statistically significant (*p*-value = 0.0294). However, no clinical significance was observed. The body weight, height, and BMI did not vary significantly (Table 2).

#### 3.1.2. Study II: Study of 30 mg of Pelubiprofen Tromethamine vs. 30 mg of Pelubiprofen

The study was explained to 53 subjects, after which screening, including vital signs, physical examination, 12-lead ECG, clinical laboratory test, etc., was performed. On the day of admission in the first period of the study, 36 subjects in total were each given a subject number. However, as two of them dropped out prior to the first administration of the investigational drug by withdrawing consent, two new subjects were recruited for replacement, so subject numbers up to 38 were used for randomizing the healthy subjects. Later, one subject after the first drug administration in the first period and two subjects after the drug administration in the second period (three subjects in total) dropped out by withdrawing consent. Hence, 36 subjects received at least one administration of the clinical trial drug, whereas 33 subjects completed the entire clinical trial (Figure 1).

The mean values of age, weight, height, and BMI were 27.53 years, 74.13 kg, 174.06 cm, and 24.44 kg/m^2^, respectively, for the 38 randomized subjects. The age, body weight, height, and BMI did not vary significantly across the subjects assigned to each sequence group (Table 2).

### 3.2. Pharmacokinetic Analysis Results

#### 3.2.1. Pharmacokinetic Pattern and Parameters in the Study of 25 mg of Pelubiprofen Tromethamine vs. 30 mg of Pelubiprofen

**Figure 2:**
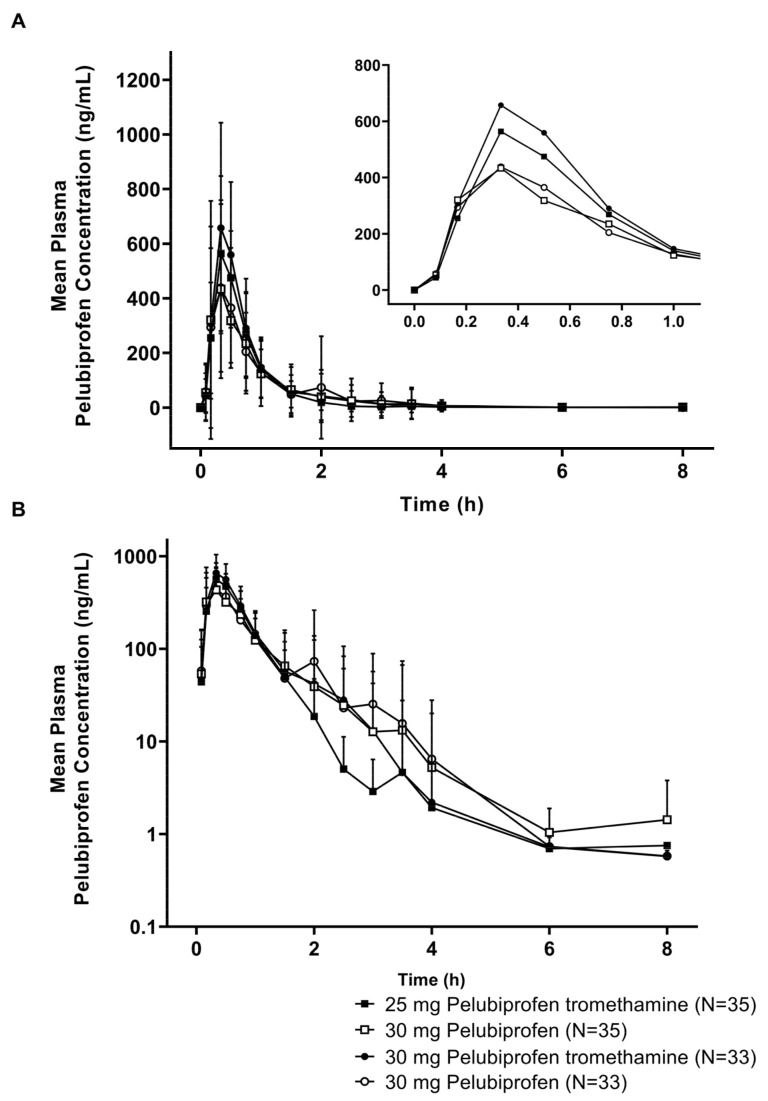
Mean plasma pelubiprofen concentration vs. time profiles after a single oral administration of 25 mg of pelubiprofen tromethamine (N = 35) or 30 mg of pelubiprofen (N = 35) in Study I and 30 mg of pelubiprofen tromethamine (N = 33) or 30 mg of pelubiprofen (N = 33) in Study II. The upper (**A**) and lower (**B**) panels are shown in linear (mean ± standard deviations) and semi-logarithmic (mean + standard deviation) scales, respectively. The figure inset within Figure 2 shows the mean plasma pelubiprofen concentration from time 0 to 1 h after dose.

Pharmacokinetic (PK) analysis was performed on the subjects exhibiting a quantifiable plasma drug concentration upon the completion of the respective blood sampling according to the schedule, after each subject had received two investigational drugs according to the protocol. The PK analysis was therefore performed on 35 subjects in Study I. The C_max_ in Study I was reached at approximately 0.33 h for both 25 mg of pelubiprofen tromethamine as the test and 30 mg of pelubiprofen as the reference (Figure 2A and Table 3). In addition, in 25 mg of pelubiprofen tromethamine and 30 mg of pelubiprofen tromethamine, the elimination pattern was similar to that of the 30 mg of pelubiprofen (Figure 2B).

For both 25 mg of pelubiprofen tromethamine and 30 mg of pelubiprofen, the median T_max_ was 0.33 h, whereas the C_max_ values were 645.80 ± 237.73 ng/mL and 619.27 ± 367.96 ng/mL, respectively, and AUC_0-t_ values 370.76 ± 91.86 h*ng/mL and 352.04 ± 102.64 h*ng/mL, respectively (Table 3).

As a result of the four period repeated crossover test of pelubiprofen, the highest within-subject variability of the reference product, 30 mg of pelubiprofen, was approximately 58% for C_max_, indicating that pelubiprofen is a highly variable drug. Therefore, the 90% CI of C_max_ in this study was extended to log 0.70–log 1.43 and the 90% CI of AUC_0-t_ to log 0.8–log 1.25 for the evaluation on the bioequivalence study criteria. The GMR (90% CI) of the C_max_ of 25 mg of pelubiprofen tromethamine was 1.16 (1.02–1.31) to fall within the log 0.70–log 1.43 range, and the GMR (90% CI) of the AUC_0-t_ was 1.07 (1.02–1.12) to fall within the log 0.8–log 1.25 range, both of which satisfied the bioequivalence study criteria.

#### 3.2.2. Pharmacokinetic Pattern and Parameters in the Study of 30 mg of Pelubiprofen Tromethamine vs. 30 mg of Pelubiprofen

Pharmacokinetic (PK) analysis was performed on the subjects exhibiting a quantifiable plasma drug concentration upon the completion of the respective blood sampling according to the schedule, after each subject had received two investigational drugs according to the protocol. The PK analysis was therefore performed on 33 subjects in Study II. In Study II, where 30 mg of pelubiprofen tromethamine and 30 mg of pelubiprofen were compared, the T_max_ values for 30 mg of pelubiprofen tromethamine as the test and 30 mg of pelubiprofen as the reference were approximately 0.33 h and 0.50 h, respectively (Figure 2A and Table 3). For 30 mg of pelubiprofen tromethamine and 30 mg of pelubiprofen, the median T_max_ values were 0.33 h and 0.50 h, respectively, whereas the C_max_ values were 770.01 ± 347.86 ng/mL and 619.47 ± 335.55 ng/mL, respectively, and the AUC_0-t_ values were 446.44 ± 123.33 h*ng/mL and 364.62 ± 107.88 h*ng/mL, respectively (Table 3).

Upon fasting, the reference pelubiprofen formulation showed the highest within-subject variability of approximately 58% for C_max_. As for Study I, the maximum within-subject variability of greater than 30% indicates that pelubiprofen is a highly variable drug. Therefore, the 90% CI of C_max_ in this study was extended to log 0.70–log 1.43 and the 90% CI of AUC_0-t_ to log 0.8–log 1.25 for the evaluation on the bioequivalence study criteria. The GMR (90% CI) of the C_max_ and AUC_0-t_ of 30 mg of pelubiprofen tromethamine were 1.29 (1.12–1.50) and 1.24 (1.18–1.30), respectively. Neither C_max_ nor AUC_0-t_ showed 90% CI within the range of log 0.8–log 1.25. As a result, neither of the PK parameters of interest did not fall within the bioequivalence study criteria.

### 3.3. Pharmacodynamic Analysis Results

#### 3.3.1. Pharmacodynamic Pattern and Parameters in the Study of 25 mg of Pelubiprofen Tromethamine vs. 30 mg of Pelubiprofen

**Figure 3:**
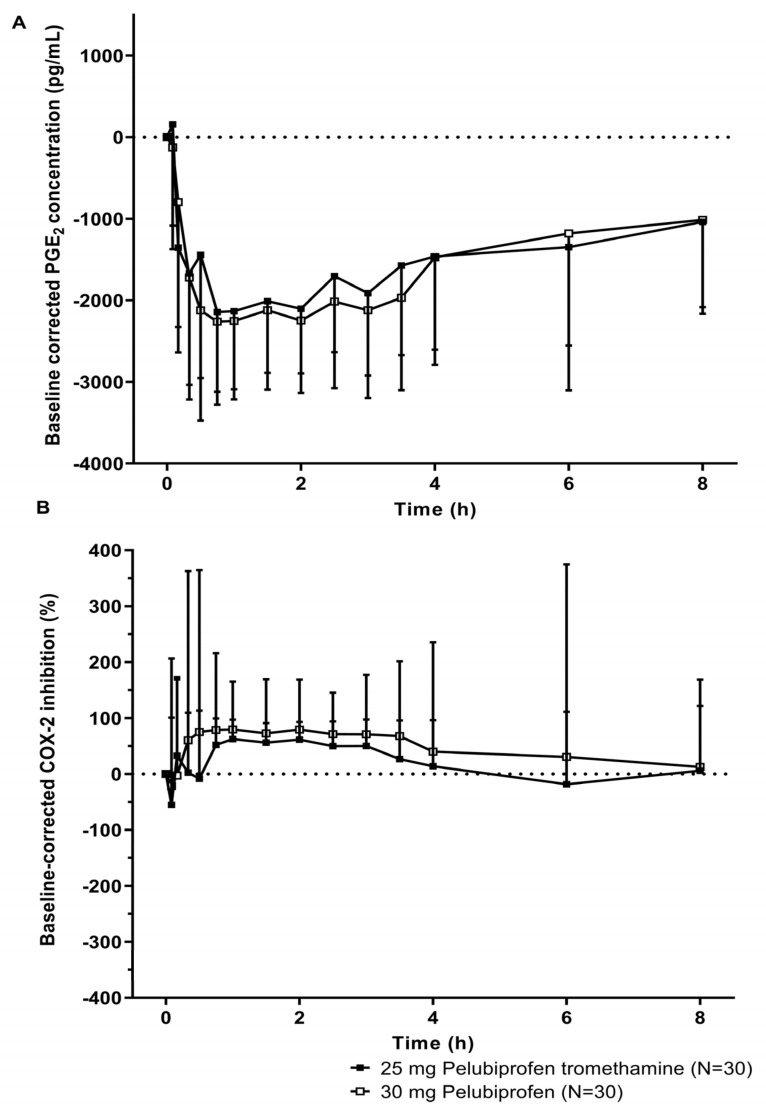
The extent of COX-2 inhibition determined from PGE_2_ concentration from baseline levels after a single oral administration of 25 mg of pelubiprofen tromethamine or 30 mg of pelubiprofen in periods 1 and 2 of Study I are shown as the baseline-corrected PGE_2_ concentration over time (**A**) and percent change from baseline COX-2 inhibition over time (**B**). The upper (**A**) and lower (**B**) panels are shown in linear scale as (mean − standard deviation) and (mean + standard deviation), respectively.

The pharmacodynamic (PD) analysis was performed on the 30 subjects in Study I, who had agreed to participate in the PD evaluation. After the administration of 25 mg of pelubiprofen tromethamine and 30 mg of pelubiprofen tromethamine, the PGE_2_ concentration decreased over time, and then a pattern of recovery was shown from around 2 h after administration. The PGE_2_ concentration was found not to vary significantly between the administration of 25 mg of pelubiprofen tromethamine and 30 mg of pelubiprofen (Figure 3A). Compared to 30 mg of pelubiprofen as the reference, the test of 25 mg of pelubiprofen tromethamine displayed a similar pattern of elimination, while a slight fall in the COX-2 inhibitory effect was observed (Figure 3B).

In Figure 3B, the COX-2 inhibitory effect appears to be slightly low for 25 mg of pelubiprofen tromethamine compared to 30 mg of pelubiprofen. However, as a result of confirming this through a statistical test, the geometric mean ratios of the maximum baseline-corrected COX-2 inhibition (I_max_) was 0.98. Thus, the COX-2 inhibition rate of 30 mg of pelubiprofen vs. 25 mg of pelubiprofen tromethamine was approximately 98%, indicating no significant difference. GMR (90% Cl) of the area under the time vs. baseline-corrected COX-2 inhibition curve from time 0 to the last measurable timepoint (AUIC) was 1.09 (0.94–1.27), and GMR (90% CI) of time of peak baseline-corrected COX-2 inhibition (T_max,i_) was 1.01 (0.75–1.35). Through this, it was confirmed that the PD of 25 mg of pelubiprofen tromethamine compared to 30 mg of pelubiprofen were similar to the PK results (Table 4).

#### 3.3.2. Pharmacodynamic Pattern and Parameters in the Study of 25 mg of Pelubiprofen Tromethamine vs. 30 mg of Pelubiprofen with the Exclusion of Outliers

The PD parameters were examined for the subjects in Study I, who had agreed to participate in the PD evaluation. The result was a markedly low level of AUC_0-t_ in certain subjects, thus necessitating the following measures. The PD data of these subjects dissatisfied normality, and therefore, the IQR of the boxplot drawn for the log-transformed area under the time vs. PGE_2_ concentration curve from time 0 to the last measurable timepoint (AUC_PGE2_) displayed two outliers (A03 and A08), for which Grubb’s test was performed. The two outliers were shown to have statistical significance.

**Figure 4:**
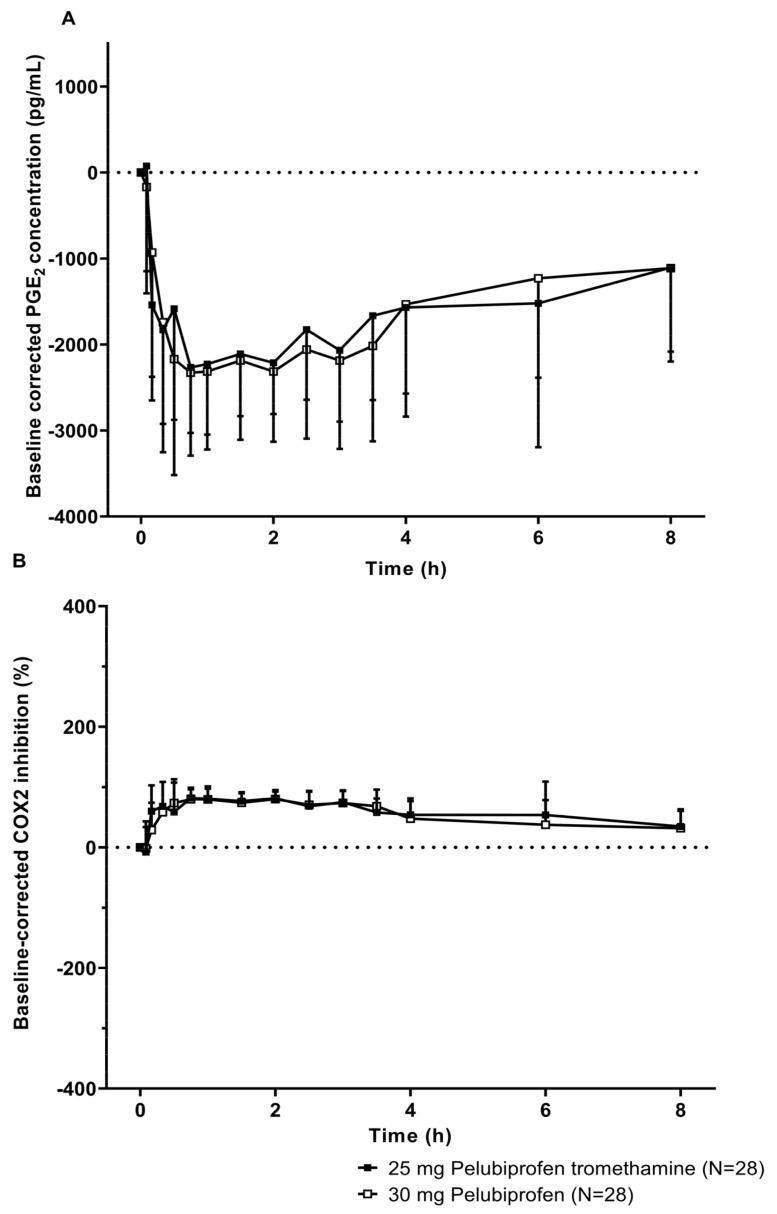
The extent of COX-2 inhibition determined from PGE_2_ concentration from baseline levels after a single oral administration of 25 mg of pelubiprofen tromethamine or 30 mg of pelubiprofen in periods 1 and 2 of Study I are shown as the baseline-corrected PGE_2_ concentration over time (**A**) and percent change from baseline COX-2 inhibition over time (**B**). The upper (**A**) and lower (**B**) panels are shown in linear scale as (mean − standard deviation) and (mean + standard deviation), respectively. Data include all subjects in period 1 and 2 without the potential outliers (A03 and A08).

Therefore, the respective subjects (A03 and A08) were excluded, and an additional PD analysis was performed on 28 subjects. The PD analysis, excluding the outliers, showed a similar PD pattern to the time all subjects were analyzed. However, Figure 4, excluding outliers confirmed that the range of mean + standard deviation, is smaller than Figure 3 including outliers. In addition, after administration of 25 mg of pelubiprofen tromethamine and 30 mg of pelubiprofen, COX-2 was rapidly suppressed, and then COX-2 inhibition was maintained until approximately 8 h (Figure 4).

The statistical analysis of the pharmacodynamic parameters in the analysis excluding the two outlier subjects (A03 and A08) showed that the COX-2 inhibitory effect was approximately 98% for 25 mg of pelubiprofen tromethamine compared to 30 mg of pelubiprofen, which did not vary greatly from the previous analysis including the outliers with the lack of statistical significance (Table 5).

### 3.4. Safety and Tolerability

#### 3.4.1. Study I: Study of 25 mg of Pelubiprofen Tromethamine vs. 30 mg of Pelubiprofen

In Study I, the safety evaluation was performed on subjects administered with the investigational drug at least once. In 3 out of 36 such subjects, 4 cases of adverse events (pruritus, n = 2; headache, n = 1; iron deficiency anemia, n = 1) were identified. In the treatment of reference, three cases of adverse events (pruritus, n = 1; headache, n = 1; iron deficiency anemia, n = 1) were identified. In the treatment of the test, one case of adverse events (pruritus, n = 1) was identified. The two cases of pruritus were found in one subject, with the evaluation indicating ‘definitely related’ in the cause–effect relationship with the investigational drug by the investigator. The one case of headache in one subject was evaluated as ‘possibly related’ with the investigational drug by the investigator. The one case of iron deficiency anemia in one subject was evaluated as ‘not related’ with the investigational drug by the investigator. The severity of all adverse events in this study was mild. 

Therefore, a total of three adverse events judged to be related to the investigational drug were confirmed (pruritus, n = 2; headache, n = 1). In one of the two pruritus cases in one subject, the symptoms disappeared without additional treatment in period I, but as the symptoms reappeared in period III, the subject had to drop out of the study as determined by the investigator. In one case of headache, symptoms were recovered without additional measures. No serious adverse events occurred during the clinical trial, and no clinically significant finding in the vital signs or 12-lead ECG, etc., was reported. The results collectively indicated that the investigational drug in this study exhibited outstanding tolerability and safety, and the level of safety upon administration did not vary from the reference.

#### 3.4.2. Study II: Study of 30 mg of Pelubiprofen Tromethamine vs. 30 mg of Pelubiprofen

In Study II, the safety evaluation was performed on subjects administered with the investigational drug at least once. In 1 out of 36 subjects, one case of adverse events (blister, n = 1) was identified, and the evaluation indicated ‘not related’ in the cause–effect relationship with the investigational drug by investigator, and the symptoms disappeared without specific treatment. In the treatment of test, one case of adverse events (blister, n = 1) was identified. The severity of these adverse events was mild, and no serious adverse events occurred during the clinical trial. No other clinically significant findings in the vital signs, 12-lead ECG, or clinical laboratory test, etc., were reported. The results collectively indicated that the investigational drug in this study exhibited outstanding tolerability and safety, and the level of safety upon administration did not vary from the reference.

## 4. Discussion

This study comprised two independent clinical trials performed in accordance with an identical randomized, open-label, oral, single-dose, fasted, two-sequence, four-period, crossover design. Pelubiprofen tromethamine, compared to pelubiprofen, showed enhanced solubility and absorption in non-clinical studies. As a result, the pelubiprofen tromethamine dose should be validated in the case of new drug development to verify whether the exposure is equivalent to 30 mg of pelubiprofen as the reference [14]. For this purpose, PK and PD characteristics of pelubiprofen and pelubiprofen tromethamine in healthy subjects were investigated in this study.

When evaluating the correlation between the PD results and the PK parameters confirmed in the 25 mg of pelubiprofen tromethamine dose group, it was found that the plasma concentration of pelubiprofen returned to baseline approximately 4 h after oral administration, whereas the COX-2 inhibition effect and the PGE_2_ concentration decrease appeared to persist up to 8 h after administration (Figure 2 and Figure 3). A reason for such deviation may come from the following PK–PD relationship. Generally, PK is evaluated with the drug concentration in the central compartment, and PD is reported to be evaluated with the drug concentration in the effect compartment, which is an effect site that directly affects the effect [15]. The drug concentration in the central compartment and the drug concentration in the effect compartment do not always coincide. In general, it shows hysteresis, in which the drug concentration in the effect compartment reaches the same concentration level at a lower concentration or at a later time than the drug concentration in the central compartment with a time difference [16]. Although the concentration at the site of effect vs. central compartment could be not always at the same levels even at steady state, given that there is sufficient time for the drugs in the central compartment to reach the effect compartment, the drug concentrations in the two compartments can be reached at the same levels. However, in the case of single dose administration, evaluating the correlation between the measured concentration and the observed effect in the central compartment due to the hysteresis effect reveals a delay in the effect vs. the concentration [15,17]. Therefore, the retention of drug effects from pelubiprofen was likely under the influence of hysteresis in this study, as pelubiprofen is a parent drug whose effects are released when the drug is converted to an active metabolite in hydroxy form in the liver.

Furthermore, in the four-period repeated crossover tests of the reference pelubiprofen 30 mg of formulation in Study I and II, the maximum within-subject variabilities of C_max_ were approximately 58%, confirming that pelubiprofen is a high-variability drug. Accordingly, the 90% Cl of the log-transformed mean of C_max_ and AUC_0-t_ was applied as log 0.70–log 1.43 and log 0.8–log1.25, respectively. As a result, compared to the reference of 30 mg of pelubiprofen, both the C_max_ and AUC_0-t_ of 25 mg of pelubiprofen tromethamine satisfied the bioequivalence study criteria. Conversely, the C_max_ and AUC_0-t_ of 30 mg of pelubiprofen tromethamine, compared to the reference, were increased by 29% and 24%, respectively, to confirm that the 90% CI fell outside the log 0.8–log 1.25 range. These results found that, compared to the reference, 30 mg of pelubiprofen tromethamine had high levels of absorption and exposure in the body (Table 3). 

In addition, COX-2-selective inhibitors exhibit antipyretic and analgesic effects by reducing the PGE_2_ concentration, and pelubiprofen as a COX-2-selective inhibitor is predicted to increase the level of COX-2 inhibition in a dose-dependent way [3,11]. However, in this study, the maximum COX-2 inhibitory effect of 25 mg of pelubiprofen tromethamine was approximately 0.98-fold compared to that of 30 mg of pelubiprofen in the PD analysis conducted on all subjects or the subjects excluding outliers (Table 4 and Table 5). In statistical terms, this indicates the lack of significant variation in PK and PD characteristics between 25 mg of pelubiprofen tromethamine and the 30 mg of pelubiprofen. Following a reduction in the drug dose, the drug exposure and effect are anticipated to drop. However, in this study, 25 mg of pelubiprofen tromethamine and 30 mg of pelubiprofen exhibited similar levels of drug exposure and effect, which is presumably attributed to the increased solubility and permeability of pelubiprofen through fusion with tromethamine as reported in previous non-clinical studies [14]. 

In line with this study, among NSAIDs, ibuprofen arginate as a salt-modified drug with enhanced absorption showed an increased level of drug exposure compared to ibuprofen. The C_max_ of ibuprofen arginate was approximately 30 mg/L and the C_max_ of ibuprofen was approximately 24 mg/L [18,19]. The improvement in drug absorption through salt modification or salt fusion is recognized in the field of pharmacology and may thus be conjectured to underlie the similar pattern observed in this study [20].

In addition, the PD evaluation in this study showed that the COX-2 inhibitory effect was substantially low in two subjects (A03 and A08) compared to other subjects and they were presented as potential outliers in Grubb’s test. Three broad factors that could have influenced the varying drug effects in the two subjects were considered: the protein binding, genetic polymorphism, and AUC. 

First, as pelubiprofen is a drug with a high protein binding rate, the fraction unbound indicates that the drug effect was low so that the unique protein abundance in each individual may have led to varying drug effects [15,21]. Second, in a previous study, celecoxib as a COX-2-selective inhibitor varied in effect owing to COX-2 genetic polymorphism, and performed in accordance with this, the COX-2 inhibitory effect could be predicted to be low in subjects with COX-2 genetic polymorphism. This was not verified in this study, however, and a follow-up study could be conducted on the COX-2 single nucleotide polymorphism (SNP) genotypes of the subjects in this study [22]. Lastly, the AUC for the drug in the two outlier subjects (A03 and A08) was lower than the mean, to which the reduced effects through low drug exposure could be attributed.

Summarily, compared to 30 mg of pelubiprofen, 25 mg of pelubiprofen tromethamine showed similar levels of drug exposure and effects with outstanding tolerability and safety. However, this study had limitations in confirming PK and PD in actual clinical settings, as it explored PK and PD by administering a single oral dose to a small number of healthy subjects. Therefore, to investigate the pain and pathophysiology of inflammatory diseases related to pelubiprofen, a greater number of patients should be recruited, and the dose should be increased to three oral administrations per day in a follow-up study reflecting actual clinical settings. This would allow detailed discussion on the gastrointestinal related adverse event comparisons between the two formulations.

## 5. Conclusions

The fusion of tromethamine salt, despite the adjustment of the pelubiprofen dose to 25 mg, was shown to lead to an exposure level that was approximately that of 30 mg of pelubiprofen. The level of COX-2 inhibition following the single dose administration of 25 mg of pelubiprofen tromethamine was similar to that of the administration of 30 mg of pelubiprofen. It is therefore predicted that 25 mg of pelubiprofen tromethamine would show no significant variation in clinical analgesic and antipyretic effects from 30 mg of pelubiprofen.

## Figures and Tables

**Table 1:** Drugs administered and parameters of analysis in both studies.

Study	Reference	Test
Pharmacokinetic analysis (C_max_, AUC_0-t_)		
Study I ^(1)^	30 mg of pelubiprofen	25 mg of pelubiprofen tromethamine
Study II ^(2)^	30 mg of pelubiprofen	30 mg of pelubiprofen tromethamine
Pharmacodynamic analysis (PGE_2_ concentration, Baseline-corrected COX-2 inhibition)		
Study I ^(1)^	30 mg of pelubiprofen	25 mg of pelubiprofen tromethamine

^(1)^ Subjects received 25 mg of pelubiprofen tromethamine and 30 mg of pelubiprofen in Study I (Study No. DW1809-102); ^(2)^ subjects received 30 mg of pelubiprofen tromethamine and 30 mg of pelubiprofen in Study II (Study No. DW1809-103). Abbreviations: C_max_, maximum concentration; AUC_0-t_, area under the time vs. concentration curve from time 0 to the last measurable timepoint; PGE_2_, Prostaglandin E_2_.

**Table 2:** (A) Demographic characteristics in Study I. (B) Demographic characteristics in Study II.

**A**					
**Demography**	**Statistics**	**Sequence A**	**Sequence B**	**Total**	***p*-Value ^(1)^**
		**N = 18**	**N = 19**	**N = 37**	
Age (years)	Mean	24.67	26.11	25.41	0.0294
	SD	7.33	6.38	6.80	
	Min	21.00	22.00	21.00	
	Max	52.00	48.00	52.00	
Weight (kg)	Mean	73.57	76.05	74.85	0.4119
	SD	10.75	9.62	10.12	
	Min	54.30	60.00	54.30	
	Max	91.50	92.20	92.20	
Height (cm)	Mean	172.32	173.72	173.04	0.4473
	SD	5.75	5.62	5.65	
	Min	162.40	163.10	162.40	
	Max	182.70	185.30	185.30	
BMI (kg/m^2^)	Mean	24.73	25.17	24.96	0.6485
	SD	2.98	2.74	2.83	
	Min	18.80	19.60	18.80	
	Max	29.90	29.50	29.90	
**B**					
**Demography**	**Statistics**	**Sequence A**	**Sequence B**	**Total**	***p*-Value ^(1)^**
		**N = 20**	**N = 18**	**N = 38**	
Age (years)	Mean	27.85	27.17	27.53	0.8031
	SD	8.24	7.37	7.75	
	Min	19.00	19.00	19.00	
	Max	50.00	42.00	50.00	
Weight (kg)	Mean	73.97	74.31	74.13	0.6823
	SD	11.75	7.83	9.95	
	Min	58.30	61.20	58.30	
	Max	96.60	87.50	96.60	
Height (cm)	Mean	173.30	174.91	174.06	0.2665
	SD	4.94	4.62	4.80	
	Min	164.80	166.00	164.80	
	Max	180.10	181.80	181.80	
BMI (kg/m^2^)	Mean	24.61	24.26	24.44	0.8378
	SD	3.48	2.08	2.87	
	Min	19.30	20.30	19.30	
	Max	29.80	28.50	29.80	

^(1)^ Wilcoxon rank-sum test.

**Table 3:** The pharmacokinetic parameters of different formulations of pelubiprofen.

Pharmacokinetic Parameters	Study I (DW1809-102) (N = 35)			Study II (DW1809-103) (N = 33)		
	25 mg Pelubiprofen Tromethamine ^(1)^	30 mg Pelubiprofen	GMR (90% CI) ^(3)^	30 mg of Pelubiprofen Tromethamine ^(2)^	30 mg Pelubiprofen	GMR (90% CI) ^(3)^
C_max_ (ng/mL)	645.80 ± 237.73	619.27 ± 367.96	1.16 (1.02–1.31)	770.01 ± 347.86	619.47 ± 335.55	1.29 (1.12–1.50)
AUC_0-t_ (ng h/mL)	370.76 ± 91.86	352.04 ± 102.64	1.07 (1.02–1.12)	446.44 ± 123.33	364.62 ± 107.88	1.24 (1.18–1.30)
AUC_0-inf_ (ng h/mL)	372.12 ± 92.16	355.25 ± 103.24	1.07 (1.02–1.12)	447.75 ± 123.40	365.71 ± 107.94	1.24 (1.18–1.30)
T_max_ (h) ^(4)^	0.33 (0.17–1.50)	0.33 (0.17–3.50)	-	0.33 (0.17–2.50)	0.50 (0.17–3.50)	-
t_1/2_ (h)	0.87 ± 0.39	0.99 ± 0.54	-	0.95 ± 0.56	0.93 ± 0.69	-
CL/F (L/h)	71.78 ± 19.79	92.12 ± 28.68	-	72.08 ± 19.97	90.55 ± 32.04	-
Vd/F (L)	87.43 ± 40.63	124.92 ± 58.82	-	94.56 ± 52.40	118.74 ± 94.09	-

Parameters are presented as the mean ± standard deviation unless otherwise stated: ^(1)^ 25 mg of pelubiprofen tromethamine and 30 mg of pelubiprofen in Study I (Study No. DW1809-102); ^(2)^ 30 mg of pelubiprofen tromethamine and 30 mg of pelubiprofen in study II (Study No. DW1809-103); ^(3)^ the expanded acceptance criterion for C_max_ of highly variable drugs with within-subject variability exceeding 50% is log 0.70~log 1.43; ^(4)^ T_max_ is presented as the median (minimum–maximum). Abbreviations: GMR, geometric mean ratios; C_max_, maximum concentration; AUC_0–t_, area under the time vs. concentration curve from time 0 to the last measurable timepoint; AUC_0-inf_, area under the time vs. concentration curve from time 0 to infinity; T_max_, time of peak plasma concentration; t1/2, terminal half-life; CL/F, apparent clearance; Vd/F, apparent volume of distribution.

**Table 4:** The COX-2 inhibition derived from PGE_2_ concentration after administration of 25 mg of pelubiprofen tromethamine and 30 mg of pelubiprofen.

Pharmacodynamic Parameters	Study I (DW1809-102) (N = 30)		Geometric Mean Ratios (90% Confidence Intervals)
	25 mg Pelubiprofen Tromethamine ^(1)^	30 mg Pelubiprofen	
PGE_2_ concentration			
C_max,PGE2_ (pg/mL)	3426.87 ± 1050.03 (30.6)	3671.70 ± 1384.24 (37.7)	-
AUC_PGE2_ (pg h/mL)	12,840.05 ± 6235.54 (48.6)	13,520.61 ± 7847.51 (58.0)	-
T_max,PGE2_ (h) ^(2)^	0.08 (0.00–8.00)	0.08 (0.00–8.00)	-
Baseline-corrected COX-2 inhibition			
I_max_ (%)	84.32 ± 20.10 (23.8)	88.14 ± 12.12 (13.8)	0.98 (0.95–1.02)
AUIC (h %)	455.54 ± 140.28 (30.8)	451.12 ± 158.65 (35.2)	1.09 (0.94–1.27)
T_max,i_ (h) ^(2)^	0.50 (0.00–3.50)	0.50 (0.33–3.50)	1.01 (0.75–1.35)

Parameters are presented as the arithmetic mean ± standard deviation (total coefficient of variation). Data include all subjects in period 1 and 2: ^(1)^ 25 mg of pelubiprofen tromethamine and 30 mg of pelubiprofen in Study I (Study No. DW1809-102); ^(2)^ T_max,PGE2_ and T_max,i_ are presented as the median (minimum–maximum). Abbreviations: PGE_2_, Prostaglandin E_2_; C_max,PGE2_, maximum PGE_2_ concentration; AUC_PGE2_, area under the time vs. PGE_2_ concentration curve from time 0 to the last measurable timepoint; T_max_,PGE_2_, time of peak PGE_2_ concentration; I_max_, geometric mean ratios of the maximum baseline-corrected COX-2 inhibition; AUIC, area under the time vs. baseline-corrected COX-2 inhibition curve from time 0 to the last measurable timepoint; Tmax,i, time of peak baseline-corrected COX-2 inhibition.

**Table 5:** The COX-2 inhibition derived from PGE_2_ concentration after administration of 25 mg of pelubiprofen tromethamine and 30 mg of pelubiprofen without potential outliers.

Pharmacodynamic Parameters	Study I (DW1809-102) (N = 28)		Geometric Mean Ratios (90% Confidence Intervals)
	25 mg Pelubiprofen Tromethamine ^(1)^	30 mg Pelubiprofen	
PGE_2_ concentration			
C_max,PGE2_ (pg/mL)	3399.74 ± 1077.09 (31.7)	3736.17 ± 1411.87 (37.8)	-
AUC_PGE2_ (pg h/mL)	13,353.29 ± 5941.05 (44.5)	13,938.40 ± 7710.14 (55.3)	-
T_max,PGE2_ (h) ^(2)^	0.08 (0.00–8.00)	0.08 (0.00–8.00)	-
Baseline-corrected COX-2 inhibition			
I_max_ (%)	87.32 ± 12.70 (14.5)	87.63 ± 12.39 (14.1)	0.98 (0.95–1.01)
AUIC (h %)	473.36 ± 114.25 (24.1)	459.54 ± 150.42 (32.7)	1.03 (0.91–1.18)
T_max,I_ (h) ^(2)^	0.50 (0.17–3.50)	0.50 (0.33–3.50)	1.00 (0.73–1.36)

Parameters are presented as the arithmetic mean ± standard deviation (total coefficient of variation). Data include all subjects in period 1 and 2 without the potential outliers (A03 and A08): ^(1)^ 25 mg of pelubiprofen tromethamine and 30 mg of pelubiprofen in Study I (Study No. DW1809-102); ^(2)^ T_max,PGE2_ and T_max,i_ are presented as the median (minimum–maximum). Abbreviations: PGE_2_, Prostaglandin E_2_; C_max,PGE2_, maximum PGE_2_ concentration; AUC_PGE2_, area under the time vs. PGE_2_ concentration curve from time 0 to the last measurable timepoint; T_max,PGE2_, time of peak PGE_2_ concentration; I_max_, geometric mean ratios of the maximum baseline-corrected COX-2 inhibition; AUIC, area under the time vs. baseline-corrected COX-2 inhibition curve from time 0 to the last measurable timepoint; T_max,i_, time of peak baseline-corrected COX-2 inhibition.

## Data Availability

The dataset supporting the conclusions of this article is included in the article.
